# Machine Learning–Based Radiomics for Differentiating Pancreatic Lesions: A Potential Tool to Enhance Clinical Decision‐Making and Nursing Management

**DOI:** 10.1155/jonm/8038903

**Published:** 2025-12-16

**Authors:** Xiaoxuan Li, Jiani Liu, Xinchi Luan, Jinfeng Cui, Xiaomin Xing, Shibo Wang, Jing Guo

**Affiliations:** ^1^ Department of Oncology, The Affiliated Hospital of Qingdao University, Qingdao, Shandong, China, qdu.edu.cn; ^2^ Department of Pharmacy, The Affiliated Hospital of Qingdao University, Qingdao, Shandong, China, qdu.edu.cn

## Abstract

**Background:**

The noninvasive diagnosis of pancreatic lesions is a critical clinical challenge. This study aims to create machine learning (ML) radiomic models for differentiating pancreatic lesions and an integrated model for pancreatic ductal adenocarcinoma (PDAC) detection.

**Methods:**

640 patients with pathologically confirmed malignant (*n* = 450), borderline (*n* = 108), or benign (*n* = 82) lesions were enrolled and divided into training (70%) and validation (30%) cohorts. Radiomic features were extracted from regions of interest on arterial and venous phase CT scans. LASSO logistic regression was used to select 36 features for building ML models, including random forest, logistic regression, support vector machine, and artificial neural networks. An integrated nomogram combining radiomic features and CA19‐9 levels was developed to distinguish PDAC from borderline tumors. Model performance was evaluated using receiver operating characteristic (ROC) curves, calibration curves, and decision curve analysis.

**Results:**

All ML models effectively differentiated the three tumor types. The random forest algorithm showed the best performance, achieving an area under the curve (AUC) of 0.99 and 0.95 in the training and validation sets, respectively. CA19‐9 was identified as an independent diagnostic factor for PDAC. The nomogram integrating radiomics and CA19‐9 achieved an AUC of 0.89 and accuracy of 0.85 in the training set, with corresponding values of 0.85 and 0.82 in the validation set.

**Conclusions:**

Radiomics‐based ML models effectively differentiated benign, borderline, and malignant pancreatic tumors. The nomogram combining radiomic features with CA19‐9 demonstrated robust performance, showing considerable potential to streamline the diagnostic process and facilitate timely care planning for patients with suspected pancreatic cancer.

## 1. Introduction

Pancreatic lesions are common in clinical practice, encompassing a broad spectrum of pathologies. Based on their biological behavior, pancreatic lesions are classified into benign, borderline, or malignant categories. Benign lesions, including serous cystic neoplasms (SCNs), pancreatic cysts, and pancreatic pseudocysts, generally carry favorable prognoses and typically do not undergo malignant transformation [1]. Conversely, malignant tumors, most commonly pancreatic ductal adenocarcinoma (PDAC), are aggressive and associated with poor prognoses [2]. Pancreatic cancer often remains asymptomatic in its early stages, resulting in delayed diagnoses and extremely low 5‐year survival rates [3]. Borderline tumors present features intermediate between benign and malignant lesions. Based on the 2006 World Health Organization classification of exocrine pancreatic tumors, mucinous cystic neoplasms (MCNs), solid pseudopapillary neoplasms (SPNs), and intraductal papillary mucinous neoplasms (IPMNs) are classified as borderline tumors [4, 5]. These tumors harbor malignant potential and may progress to malignancy if not adequately treated [6, 7]. The prognoses for different pancreatic lesions vary significantly, necessitating distinct therapeutic strategies. However, the imaging manifestations of these lesions can be similar [8]. Conventional diagnostic methods face challenges in definitively characterizing pancreatic lesions, particularly in differentiating borderline tumors from malignant tumors.

Histopathologic biopsy remains the gold standard for definitive diagnosis. However, the pancreas’s intricate anatomy poses technical challenges and increases risks such as hemorrhage, infection, and tumor seeding [9, 10]. Conventional imaging modalities, including ultrasonography, computed tomography (CT), and magnetic resonance imaging (MRI), have inherent limitations in detecting pancreatic lesions and accurately differentiating benign from malignant tumors. For example, preoperative imaging achieves a diagnostic accuracy of only 33.0% in distinguishing subtypes of pancreatic cystic neoplasms [11]. The widely used serum tumor marker carbohydrate antigen 19‐9 (CA19‐9) exhibits suboptimal sensitivity, reported at approximately 65% for resectable pancreatic cancer [12]. From a nursing management perspective, diagnostic delays and the risks associated with invasive procedures translate to increased patient anxiety, potential for complications requiring intensive nursing care, and a greater burden on healthcare resources.

The advancements in machine learning (ML) and radiomics offer promising solutions to these diagnostic hurdles. Radiomics, a high‐throughput computational approach, enables the extraction and quantitative analysis of extensive imaging‐derived features from radiological data [13]. This methodology can reveal subtle differences not discernible through conventional imaging. Commonly used ML models include logistic regression (LR), support vector machines (SVMs), random forest (RF), gradient boosting, and artificial neural networks (ANNs) [14, 15]. The integration of radiomics and ML has been shown to play a significant role in the early diagnosis, prognosis prediction, and therapeutic response evaluation of pancreatic tumors [16–18].

The objective of this study is to develop radiomics‐based ML models to distinguish benign, borderline, and malignant pancreatic tumors. While most existing research focuses on differentiating pancreatic lesions from normal tissue or between two lesion types, few have addressed the simultaneous classification of three categories. Furthermore, for nonbenign pancreatic tumors, we aim to construct a combined diagnostic model integrating radiomics and CA19‐9 levels to improve diagnostic accuracy. This integrated model is designed to provide a potential tool for nurse managers and clinical teams to streamline the diagnostic process, enhance patient education, and facilitate timely care planning for individuals with suspected pancreatic cancer.

## 2. Materials and Methods

### 2.1. Patients

This retrospective study included patients who were pathologically diagnosed with pancreatic tumors following surgery between January 2015 and December 2021. This study was approved by the ethics committee of the Affiliated Hospital of Qingdao University (Reference No: QYFY WZLL 28748) and conducted in accordance with the World Medical Association Declaration of Helsinki. Given the retrospective nature of the study, the requirement for informed consent was waived.

**Figure 1:**
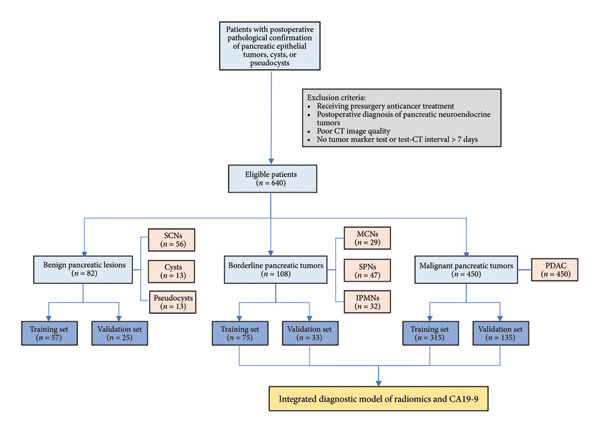
Flowchart shows patient selection. CT, computed tomography; SCNs, serous cystic neoplasms; MCNs, mucinous cystic neoplasms; SPNs, solid pseudopapillary neoplasms; IPMNs, intraductal papillary mucinous neoplasms; PDAC, pancreatic ductal adenocarcinoma; CA19‐9, carbohydrate antigen 19‐9.

The inclusion criteria were as follows: (i) postoperative pathological confirmation of pancreatic epithelial tumors, cysts, or pseudocysts; (ii) preoperative upper abdominal contrast‐enhanced CT examination with satisfactory image quality; and (iii) tumor markers tested within 7 days prior to the contrast‐enhanced CT scan. Exclusion criteria included the following: (i) receiving any anticancer treatment before surgery; (ii) postoperative pathological diagnosis of pancreatic neuroendocrine tumors (PNETs); (iii) inadequate CT image quality, including the presence of artifacts or images unsuitable for segmentation analysis; and (iv) absence of tumor marker testing or an interval exceeding 7 days between the tumor marker test and the CT examination. The cohort was randomly divided into a training set and a validation set in a 7:3 ratio. Figure 1 shows the flow of patient selection.

Clinical characteristics collected in this study included patient gender, age, smoking status, history of diabetes, tumor location, and serum tumor marker levels. Serum tumor marker levels were quantified using the ELISA method. Test results were considered elevated if the serum CA19‐9 level was ≥ 37 u/mL, carbohydrate antigen 12‐5 (CA12‐5) was ≥ 35 u/mL, or carcinoembryonic antigen (CEA) was ≥ 5 ng/mL.

### 2.2. CT Acquisition

All patients met the surgical indications and were in accordance with recent European recommendations [19, 20]. All CT examinations were performed prior to surgical resection. Contrast‐enhanced CT scans of the upper abdomen were conducted using 64‐slice spiral CT scanners (Somatom Sensation 64, Siemens Healthcare; Discovery 750, GE Healthcare) with the following settings: a tube voltage of 120 kVp, a tube current of 210 mAs, a detector collimation of 64 × 0.625 mm, a beam pitch of 1.375, and a gantry rotation time of 0.5 s. The section thickness was 2.5 mm, and the image size was 512 × 512 pixels. All examinations included both arterial and venous phase acquisitions. The arterial and venous phase images were obtained at 25s and 60 s, respectively, after intravenous administration of the contrast agent at a dose of 2 mL/kg, delivered at a rate of 3.0 mL/s.

### 2.3. Postoperative Pathology Acquisition

All pancreatic tumor specimens were meticulously standardized by experienced pathologists. The tumor classification was determined in strict accordance with the WHO Classification of Digestive System Tumors, 5th Edition (2019). The pathological findings were further reviewed and validated by senior experts with high qualifications before the final postoperative pathological reports were issued.

### 2.4. CT Image Segmentation and Preprocessing

The image assessment was independently performed by two experienced abdominal radiologists with 7 and 20 years of expertise, respectively. For challenging cases, an additional senior radiologist specializing in pancreatic tumors with over 20 years of expertise provided further review. All analyses were conducted blinded to clinical and pathological data, with radiologists evaluating both arterial and venous phase CT images to assess the following morphological features: tumor location and margin, maximum tumor diameter on axial CT, calcification, cystic lesions, region of interest (ROI), and CT values.

Tumor segmentation was performed using the open‐source 3D Slicer platform (v4.10.2, https://www.slicer.org) with semiautomated/manual hybrid delineation. Semiautomated tools employing adaptive thresholding brushes minimized manual corrections by excluding voxels exceeding preset intensity thresholds. For multifocal lesions, dominant pancreatic masses were selected based on surgical planning criteria. All contours were manually refined on axial CT slices by radiologists blinded to clinical outcomes, meticulously avoiding adjacent normal tissues and vasculature to generate ROIs. A representative example of the manual ROI delineation is shown in Figure 2.

Figure 2A representative example of the manual ROI delineation. ROI, region of interest.

**Figure:**
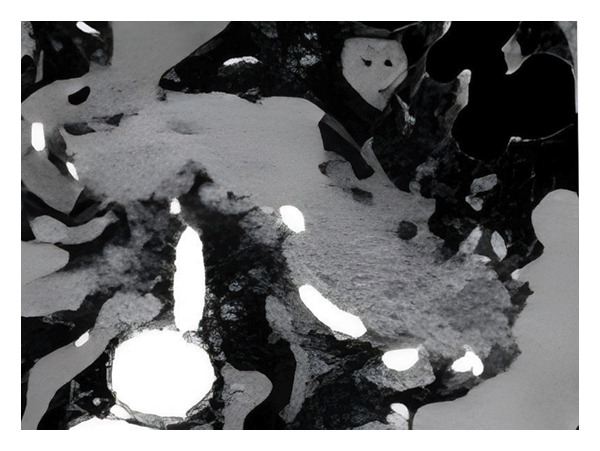
(a)

**Figure:**
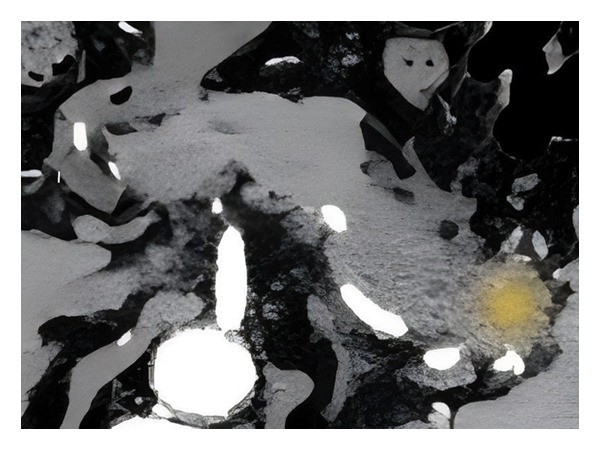
(b)

### 2.5. Radiomic Feature Extraction and Selection

A total of 112 pancreatic radiomic features were computed and extracted from ROIs using the feature extractor algorithm of the Python package PyRadiomics (https://github.com/radiomics/pyradiomics). These features encompassed shape, first‐order, gray‐level co‐occurrence matrix (GLCM), gray‐level dependence matrix (GLDM), gray‐level run‐length matrix (GLRLM), gray‐level size zone matrix (GLSZM), and neighborhood gray tone difference matrix (NGTDM). The extracted radiomic features adhered to the Image Biomarker Standardization Initiative (IBSI) guidelines [21].

The dimensionality reduction and selection of radiomic features in the training set were carried out as follows: First, one‐way analysis of variance (ANOVA) was used to identify radiomic features exhibiting significant differences across the three groups. Subsequent dimensionality reduction was performed using the Least Absolute Shrinkage and Selection Operator (LASSO) regression model. To further reduce the risk of overfitting, tenfold cross‐validation was employed to identify the value of the penalty parameter lambda (*λ*) that minimizes model error. Features with nonzero coefficients were defined as weights, and these features were ranked based on their weights to identify high‐value, nonredundant, and informative candidate features for subsequent radiomic model development. Finally, the radiomics score (Rad‐Score) for each patient was calculated using a linear combination of the selected radiomic features and their corresponding weight coefficients.

### 2.6. Model Construction and Evaluation

This study employed four ML algorithms—RF, LR, SVM, and ANN—to develop radiomic models. The diagnostic performance of these models was evaluated by the area under the curve (AUC) of receiver operating characteristic (ROC), along with sensitivity, specificity, and accuracy. To further distinguish PDAC from borderline tumors, we constructed a nomogram by combining the Rad‐Score with CA19‐9 levels. The resulting nomogram score directly reflects the effectiveness of this differentiation. The nomogram’s diagnostic performance was quantified via ROC analysis in both the training and validation cohorts. Its clinical utility was further evaluated using decision curve analysis (DCA) and calibration curves.

### 2.7. Statistical Analysis

Statistical analyses were performed using IBM SPSS Statistics 20.0 (IBM Corp., Armonk, NY, USA) and Python 3.7 (Python Software Foundation, Wilmington, DE). Quantitative data are presented as the mean ± standard deviation. ANOVA was used to evaluate age differences across all three groups, while the *T*‐test was applied to compare age differences between the malignant and borderline groups. For the remaining clinical characteristics, chi‐square tests were utilized to identify potential disparities. F represents the variance value derived from ANOVA, and *χ* ^2^ denotes the Pearson chi‐square value. No missing data were present in the final dataset for the variables analyzed. A *p* value of < 0.05 was considered statistically significant.

## 3. Results

### 3.1. Clinical Characteristics

Our study included 640 patients who were postoperatively confirmed to have pancreatic epithelial tumors, cysts, or pseudocysts. Subjects were categorized into three groups: 450 cases of malignant pancreatic tumors (all confirmed as PDAC), 108 cases of borderline tumors, and 82 cases of benign lesions. Among the borderline tumors, there were 29 cases of MCNs, 47 cases of SPNs, and 32 cases of IPMNs. The benign lesions included 56 cases of SCNs, 13 cases of pancreatic cysts, and 13 cases of pancreatic pseudocysts.

The baseline characteristics of the three groups are presented in Table 1. In all groups, the proportion of males was relatively high, at 73.8% in the malignant group, 71.3% in the borderline group, and 69.5% in the benign group. A high percentage of patients were smokers, at 64.4%, 56.5%, and 64.6%, respectively. Lesions located in the head and uncinate process of the pancreas were more common, occurring in 70.9%, 63.9%, and 67.1% of cases, respectively. Over half of the patients in each group exhibited elevated preoperative CA12‐5 and CEA levels. There were no significant differences in age, gender, smoking status, history of diabetes, tumor location, or preoperative CA12‐5 and CEA levels among the groups. In contrast, a significant difference in preoperative CA19‐9 levels was observed, with 87.6% of patients in the malignant group demonstrating elevated levels.

**Table 1:** Baseline characteristics of the study population.

Characteristic	Malignant tumors (*n* = 450)	Borderline tumors (*n* = 108)	Benign lesions (*n* = 82)	F/*χ* ^2^	*P* value
Age (years, mean ± SD)	67.94 ± 11.41	64.89 ± 11.13	63.52 ± 11.67	1.168	0.13
Sex [*n* (%)]				0.788	0.67
Male	332 (73.8%)	77 (71.3%)	57 (69.5%)		
Female	118 (26.2%)	31 (28.7%)	25 (30.5%)		
Smoking status [*n* (%)]				2.465	0.292
Yes	290 (64.4%)	61 (56.5%)	53 (64.6%)		
No	160 (35.6%)	47 (43.5%)	29 (35.4%)		
History of diabetes [*n* (%)]				0.423	0.809
Yes	128 (28.4%)	30 (27.8%)	20 (24.4%)		
No	322 (71.6%)	78 (72.2%)	62 (75.6%)		
Tumor location [*n* (%)]				2.206	0.332
Head of pancreas, uncinate process	319 (70.9%)	69 (63.9%)	55 (67.1%)		
Body, tail of pancreas	131 (29.1%)	39 (36.1%)	27 (32.9%)		
Preoperative CA19‐9 [*n* (%)]				211.013	< 0.001
≥ 37u/mL	394 (87.6%)	45 (41.7%)	16 (19.5%)		
< 37u/mL	56 (12.4%)	63 (58.3%)	66 (80.5%)		
Preoperative CA12‐5 [*n* (%)]				0.659	0.719
≥ 35u/mL	294 (65.3%)	75 (69.4%)	54 (65.9%)		
< 35u/mL	156 (34.7%)	33 (30.6%)	28 (34.1%)		
Preoperative CEA [*n* (%)]				1.380	0.501
≥ 5 ng/mL	292 (64.9%)	65 (60.2%)	49 (59.8%)		
< 5 ng/mL	158 (35.1%)	43 (39.8%)	33 (40.2%)		

Abbreviations: CA12‐5, carbohydrate antigen 12‐5; CA19‐9, carbohydrate antigen 19‐9; CEA, carcinoembryonic antigen; SD, standard deviation.

### 3.2. Feature Screening and Radiomic Signature Establishment

Following segmentation, preprocessing, feature extraction, and normalization, a high‐dimensional data space was formed. Variable analysis was conducted on the training set, and a total of 112 features, including shape features, first‐order statistics, GLCM, GLDM, GLRLM, GLSZM, and NGTDM, were extracted using the pyradiomics package. LASSO regression was applied to select from the 112 radiomic features, with tenfold cross‐validation employed to determine the optimal penalty term *λ*. The model error was minimized when log(*λ*) was −4.58 (Figures 3(a) and 3(b)). The features were ranked based on the weight coefficients derived from the LASSO regression to assess their impact on predictive outcomes. A total of 36 radiomic features showed significant differences among the three categories. Figure 3(c) reveals that features with larger weight coefficients were predominantly shape features (e.g., elongation and axis lengths) and first‐order statistics (e.g., total energy and skewness), while texture features had lower weights. These findings suggest that the global tumor morphology and overall signal distribution contribute more strongly to classification than fine‐grained texture descriptors. The low contribution of texture features may be related to the fact that pancreatic lesions often lack overt heterogeneity compared with other solid tumors. The detailed feature coefficients are presented in Table 2. These features were linearly combined after being weighted by their respective coefficients to construct the Rad‐Score. The detailed formula of the Rad‐Score is shown in Appendix 1.

Figure 3LASSO coefficients of radiomic features. (a) Distribution of LASSO coefficients for the 112 radiomic features. The optimal *λ* was selected using tenfold cross‐validation, resulting in 36 radiomic features with nonzero coefficients. (b) LASSO coefficient profiles demonstrating the shrinkage of regression coefficients to exactly zero with increasing *λ* penalty. (c) Histogram of the Rad‐Score illustrating the final 36 imaging features arranged by their relative importance. LASSO, Least Absolute Shrinkage and Selection Operator; Rad‐Score, radiomics score.

**Figure:**
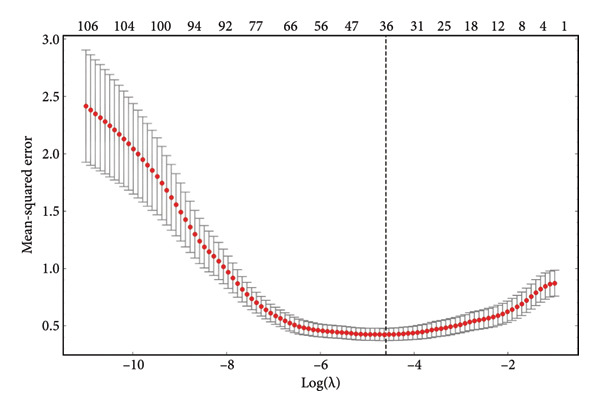
(a)

**Figure:**
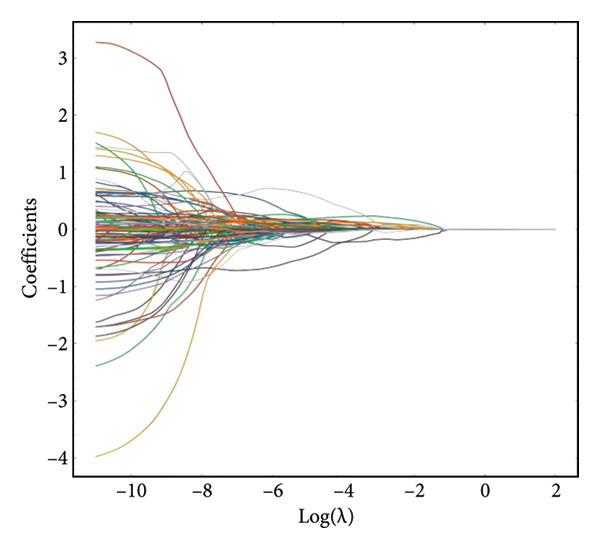
(b)

**Figure:**
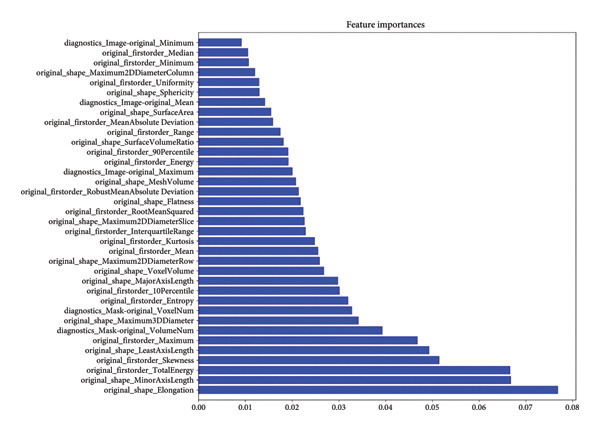
(c)

**Table 2:** 36 selected CT radiomic features and their corresponding weight coefficients.

CT radiomic features	Weight coefficients
diagnostics_image‐original_mean	0.017903666
diagnostics_image‐original_Minimum	0.017882765
original_shape_major Axis Length	0.026203535
original_shape_Maximum2DDiameterRow	−0.054928113
original_shape_Maximum3DDiameter	−0.007444548
original_shape_Sphericity	0.199637919
original_shape_Surface Volume Ratio	0.167943896
original_firstorder_10 Percentile	0.020732002
original_firstorder_Energy	0.094331627
original_firstorder_Kurtosis	−0.006846541
original_firstorder_maximum	0.041875133
original_firstorder_Skewness	0.001320465
original_glcm_Cluster Shade	−0.021916361
original_glcm_Correlation	0.071006008
original_glcm_Idm	−0.180484748
original_glcm_Idmn	0.096774879
original_glcm_Idn	0.057963618
original_glcm_Imc1	0.108379175
original_glcm_Inverse Variance	0.106859878
original_glcm_Joint Energy	−0.114233203
original_gldm_Dependence Entropy	−0.393573913
original_gldm_Large Dependence Low Gray‐Level Emphasis	0.1104212
original_glrlm_Gray‐Level Non Uniformity Normalized	−0.053043034
original_glrlm_Run Entropy	0.532133203
original_glrlm_Run Variance ‐	0.031499362
original_glszm_Gray‐Level Non Uniformity	−0.024699234
original_glszm_Gray‐Level Non Uniformity Normalized	0.035733763
original_glszm_Large Area Emphasis	0.008456957
original_glszm_Large Area Low Gray‐Level Emphasis	−0.035387708
original_glszm_Size Zone Non Uniformity Normalized	0.070738022
original_glszm_Small Area Low Gray‐Level Emphasis	−0.027782604
original_glszm_Zone Variance	0.006126049
original_ngtdm_Busyness	0.126837444
original_ngtdm_Coarseness	0.013006386
original_ngtdm_Complexity	−0.003067003
original_ngtdm_Strength	−0.061213837

Abbreviation: CT, computed tomography.

### 3.3. Performance Comparison of Radiomic Models

Based on the 36 selected radiomic features, we recast the original three‐class problem into three binary classification tasks: Class 0 distinguished benign from nonbenign lesions, Class 1 differentiated borderline tumors with benign and malignant lesions, and Class 2 separated adenocarcinomas from nonadenocarcinomas. Predictive models were subsequently developed using RF, LR, SVM, and ANN algorithms. In the training set, each model exhibited excellent discriminatory performance. The AUC values were 0.99 (95% CI, 0.98–0.99) for the RF model, 0.98 (95% CI, 0.97–0.99) for LR, 0.97 (95% CI, 0.96–0.98) for SVM, and 0.99 (95% CI, 0.98–0.99) for ANN. The RF model exhibited superior discriminative performance, with an accuracy of 99.8%, sensitivity of 99.2%, and specificity of 99.5%. The LR model achieved an accuracy of 96.6%, a sensitivity of 96.5%, and a specificity of 94.3%. The SVM model yielded an accuracy of 92.5%, a sensitivity of 95.6%, and a specificity of 83.9%. The ANN model performed comparably to the RF model, with an accuracy of 99.5%, sensitivity of 99.2%, and specificity of 99.4% (Figure 4). In the validation cohort, the RF model retained the highest AUC (0.95; 95% CI, 0.94–0.97), with an accuracy of 93.1%, sensitivity of 97.2%, and specificity of 90.2%. The LR model achieved an AUC of 0.93 (95% CI, 0.92–0.95), with 92.1% accuracy, 90.5% sensitivity, and 92.7% specificity. The SVM showed an AUC of 0.94 (95% CI, 0.93–0.97), an accuracy of 91.1%, sensitivity of 93.5%, and specificity of 85.3%. The ANN model demonstrated an AUC of 0.93 (95% CI, 0.92–0.96), with 92.1% accuracy, 90.7% sensitivity, and 95.1% specificity (Figure 5).

Figure 4ROC curves of the radiomic model in distinguishing benign, borderline, and malignant pancreatic tumors based on the training set for (a) random forest, (b) logistic regression, (c) support vector machine, and (d) artificial neural network. Class 0 distinguishes benign from nonbenign lesions. Class 1 differentiates borderline tumors from benign and malignant lesions. Class 2 separates adenocarcinomas from nonadenocarcinomas. ROC, receiver operating characteristic.

**Figure:**
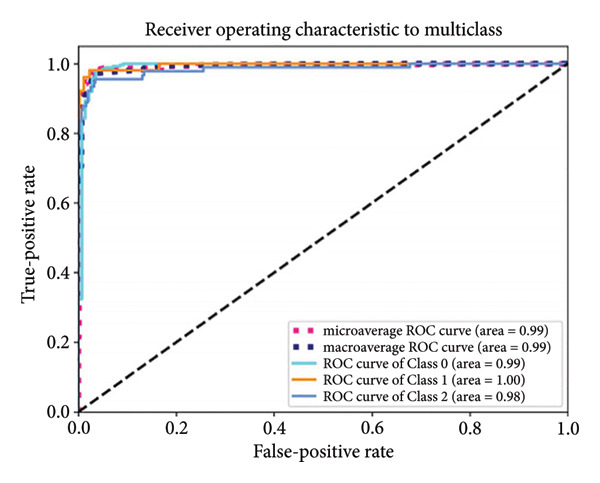
(a)

**Figure:**
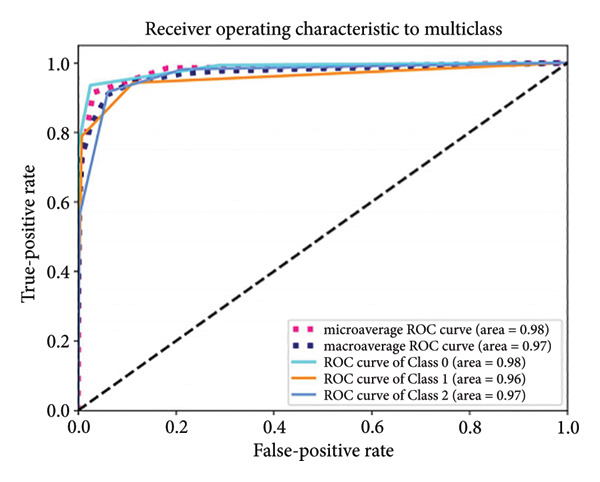
(b)

**Figure:**
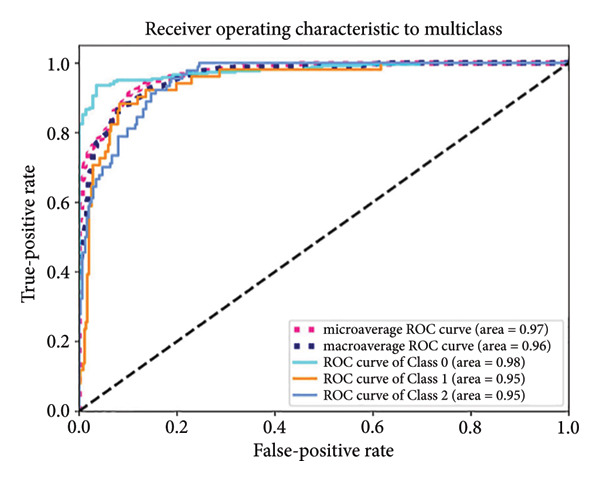
(c)

**Figure:**
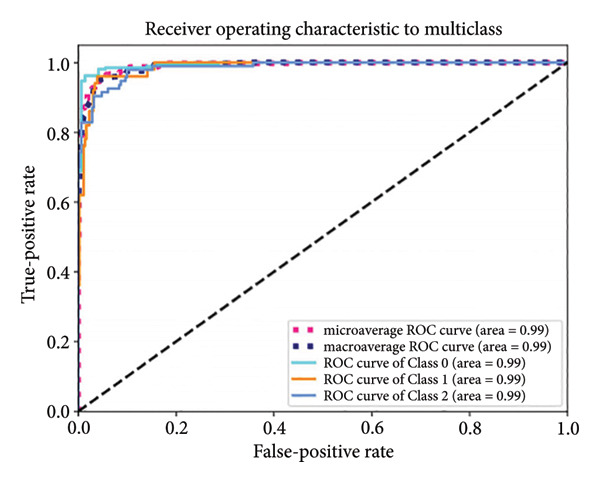
(d)

Figure 5ROC curves of the radiomic model in distinguishing benign, borderline, and malignant pancreatic tumors based on the validation set for (a) random forest, (b) logistic regression, (c) support vector machine, and (d) artificial neural network. Class 0 distinguishes benign from nonbenign lesions. Class 1 differentiates borderline tumors from benign and malignant lesions. Class 2 separates adenocarcinomas from nonadenocarcinomas. ROC, receiver operating characteristic.

**Figure:**
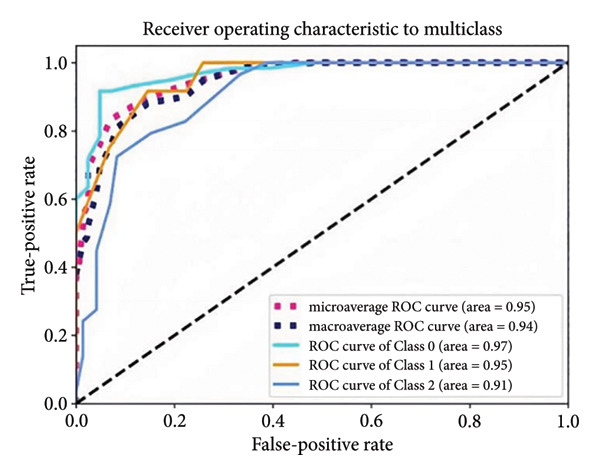
(a)

**Figure:**
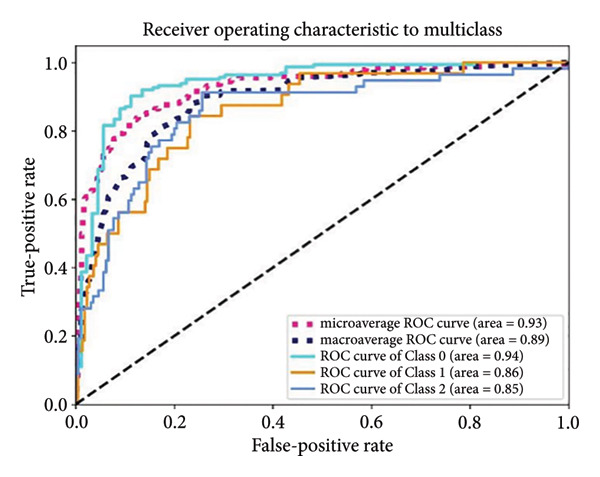
(b)

**Figure:**
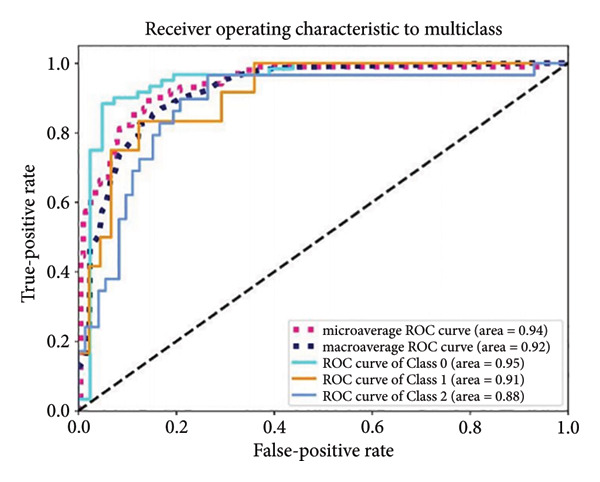
(c)

**Figure:**
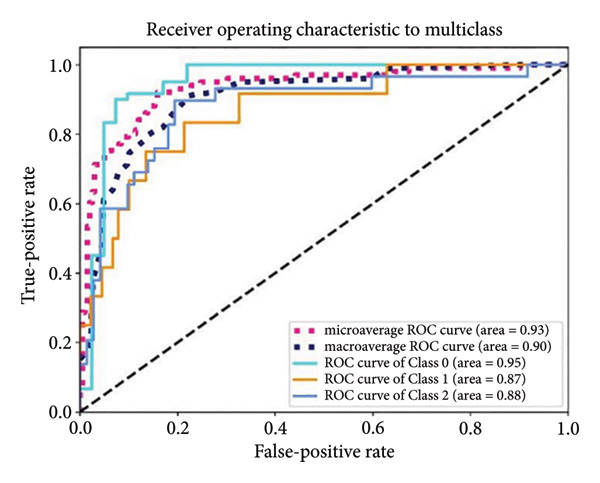
(d)

A summary of the diagnostic performance across these models is provided in Table 3. Overall, all models exhibit high discriminatory power, with the RF model demonstrating the most robust diagnostic performance with superior AUC, accuracy, specificity, and sensitivity across both training and validation sets.

**Table 3:** Comparison of diagnostic performance of the radiomic model in the training and validation cohorts.

Algorithm	Group	AUC (95% Cl)	Accuracy	Sensitivity	Specificity
Random forest	Training set	0.99 (0.98‐0.99)	0.998	0.992	0.995
	Validation set	0.95 (0.94‐0.97)	0.931	0.972	0.902
Logistic regression	Training set	0.98 (0.97‐0.99)	0.966	0.965	0.943
	Validation set	0.93 (0.92‐0.95)	0.921	0.905	0.927
Support vector machine	Training set	0.97 (0.96‐0.98)	0.925	0.956	0.839
	Validation set	0.94 (0.93‐0.97)	0.911	0.935	0.853
Artificial neural network	Training set	0.99 (0.98‐0.99)	0.995	0.992	0.994
	Validation set	0.93 (0.92‐0.96)	0.921	0.907	0.951

Abbreviations: AUC, area under the curve; CI, confidence interval.

### 3.4. Development and Performance of the Combined Nomogram

Discriminating PDAC from borderline tumors is particularly challenging. To enable a noninvasive diagnosis of PDAC, we analyzed the baseline characteristics of patients with PDAC and those with borderline tumors. Our analysis revealed no statistically significant differences between the two groups with respect to age, gender, smoking status, diabetes history, tumor location, or preoperative CA12‐5 and CEA levels. Notably, preoperative CA19‐9 levels were significantly elevated in patients with PDAC compared to those with tumors of malignant potential (Table 4), identifying preoperative CA19‐9 as an independent diagnostic factor for PDAC. These findings suggest that elevated preoperative CA19‐9 levels may strongly correlate with a histopathologic diagnosis of PDAC.

**Table 4:** Comparison of characteristics in malignant and borderline pancreatic tumors in the training and validation set.

Characteristic	Training set (*n* = 390)		*P* value	Validation set (*n* = 168)		*P* value
	Malignant tumors (*n* = 315)	Borderline tumors (*n* = 75)		Malignant tumors (*n* = 135)	Borderline tumors (*n* = 33)	
Age (years, mean ± SD)	67.65 ± 11.06	64.24 ± 11.35	0.621	68.01 ± 11.68	65.85 ± 11.43	0.714
Sex [*n* (%)]			0.742			0.125
Male	225 (71.4%)	55 (73.4%)		107 (79.3%)	22 (66.7%)	
Female	90 (28.6%)	20 (22.6%)		28 (20.7%)	11 (33.3%)	
Smoking status [*n* (%)]			0.249			0.279
Yes	195 (61.9%)	41 (54.7%)		95 (70.4%)	20 (60.6%)	
No	120 (38.1%)	34 (45.3%)		40 (29.6%)	13 (39.4%)	
History of diabetes [*n* (%)]			0.682			0.394
Yes	85 (27.0%)	22 (29.3%)		43 (31.9%)	8 (24.2%)	
No	230 (73.0%)	53 (70.7%)		92 (68.1%)	25 (75.8%)	
Tumor location [*n* (%)]			0.125			0.68
Head of pancreas, uncinate process	228 (72.4%)	48 (64.0%)		91 (67.4%)	21 (63.6%)	
Body, tail of pancreas	87 (27.6%)	27 (36.0%)		44 (32.6%)	12 (36.4%)	
Preoperative CA19‐9 [*n* (%)]			< 0.001			< 0.001
≥ 37u/mL	280 (88.9%)	32 (42.7%)		114 (84.4%)	13 (39.4%)	
< 37u/mL	35 (11.1%)	43 (57.3%)		21 (15.6%)	20 (60.6%)	
Preoperative CA12‐5 [*n* (%)]			0.124			0.355
≥ 35u/mL	197 (62.5%)	54 (72.0%)		97 (71.9%)	21 (63.6%)	
< 35u/mL	118 (37.5%)	21 (28.0%)		38 (28.1%)	12 (36.4%)	
Preoperative CEA [*n* (%)]			0.073			0.304
≥ 5 ng/mL	211 (67.0%)	42 (56.0%)		81 (60%)	23 (69.7%)	
< 5 ng/mL	104 (33.0%)	33 (44.0%)		54 (40.0%)	10 (30.3%)	

Abbreviations: CA12‐5, carbohydrate antigen 12‐5; CA19‐9, carbohydrate antigen 19‐9; CEA, carcinoembryonic antigen; SD, standard deviation.

Furthermore, we integrated preoperative CA19‐9 levels with the Rad‐Score in the training set to construct a nomogram for distinguishing PDAC from pancreatic borderline tumors. This model was visualized using a nomogram (Figure 6(a)). To use the nomogram, locate the patient’s CA19‐9 level and Rad‐Score, sum the corresponding points, and map to the probability scale to estimate the risk of PDAC. DCA curves demonstrated that patient management based on our model (i.e., diagnosing PDAC) provided a greater net benefit compared to strategies of treating all patients or none (Figures 6(b) and 6(c)). Calibration curves in both the training and validation sets confirmed the model’s good fit (Figures 6(d) and 6(e)). The model demonstrated robust diagnostic performance across both cohorts. In the training set, the model achieved an AUC of 0.89, with accuracy, sensitivity, and specificity of 0.85, 0.94, and 0.87, respectively. These metrics remained stable in the validation set, with corresponding values of 0.85, 0.82, 0.92, and 0.83 (Table 5, Figures 6(f) and 6(g)). These findings indicate that the nomogram based on radiomic features and CA19‐9 demonstrates robust diagnostic performance for PDAC across both cohorts. The integration of biological markers and radiomic features may provide a clinically interpretable tool for personalized diagnostic decision‐making.

Figure 6Development and performance of the radiomics and CA19‐9 combined diagnostic model for differentiating PDAC from borderline pancreatic tumors. (a) Nomogram developed to predict the probability of PDAC. DCA curves for the training set (b) and validation set (c). The red line represents the diagnostic model’s results; the solid black line indicates intervention for all samples (diagnosing all as PDAC); the dashed black line represents no intervention for any samples. Calibration curves for the training set (d) and validation set (e). ROC curves for the training set (f) and validation set (g). CA19‐9, carbohydrate antigen 19‐9; DCA, decision curve analysis; ROC, receiver operating characteristic.

**Figure:**
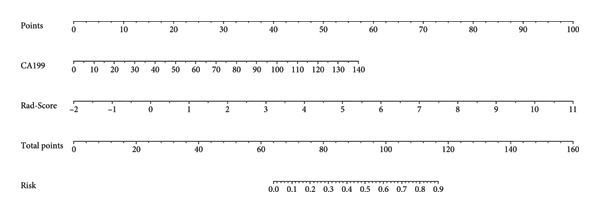
(a)

**Figure:**
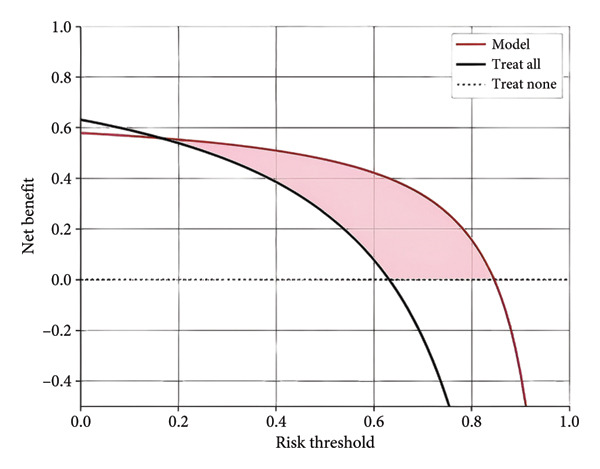
(b)

**Figure:**
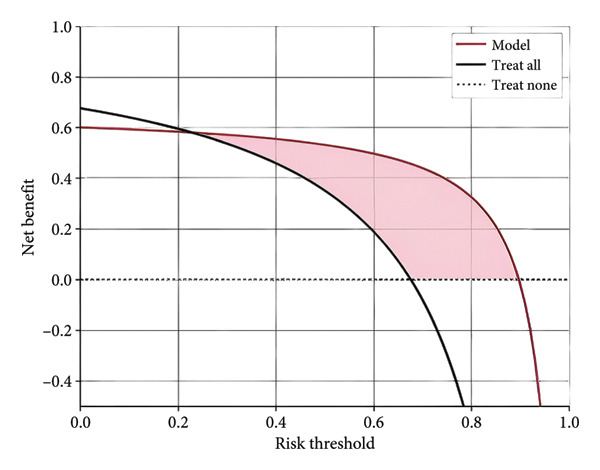
(c)

**Figure:**
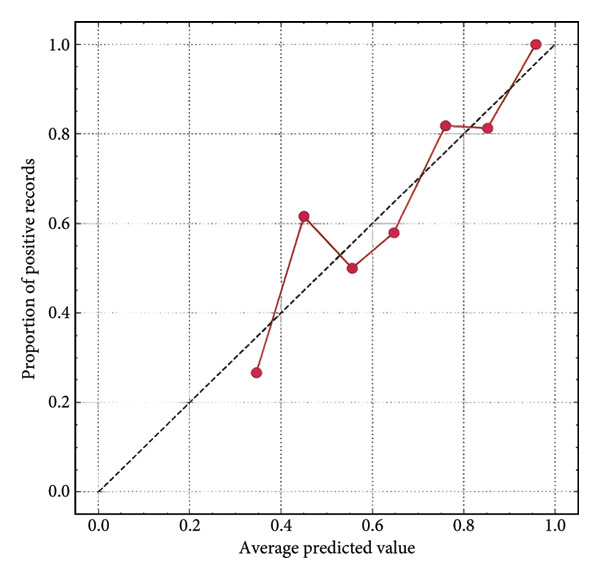
(d)

**Figure:**
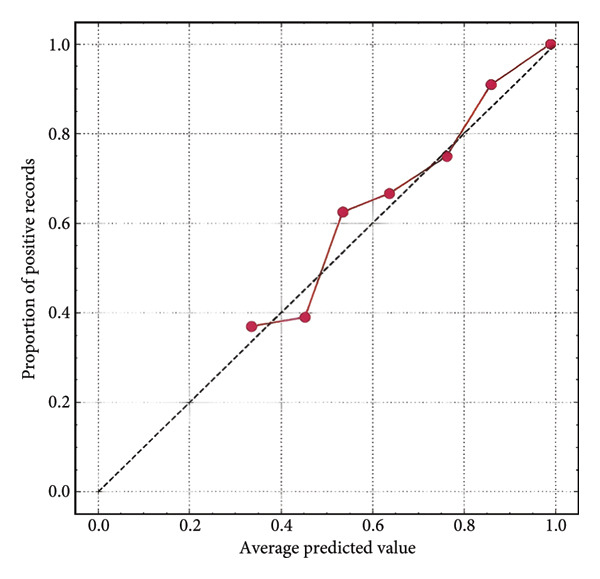
(e)

**Figure:**
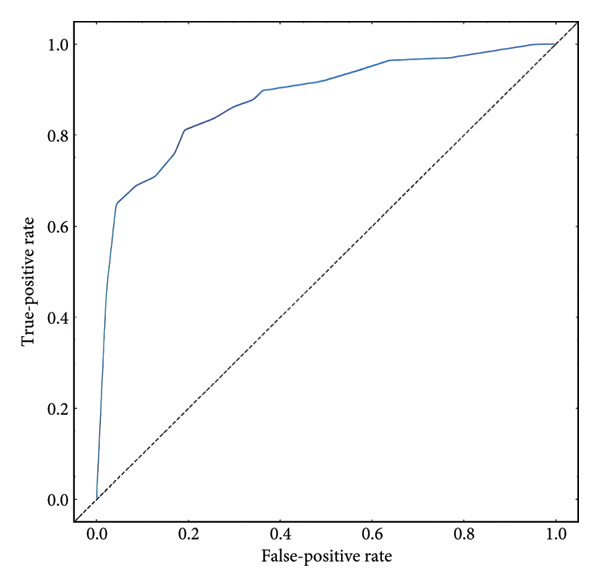
(f)

**Figure:**
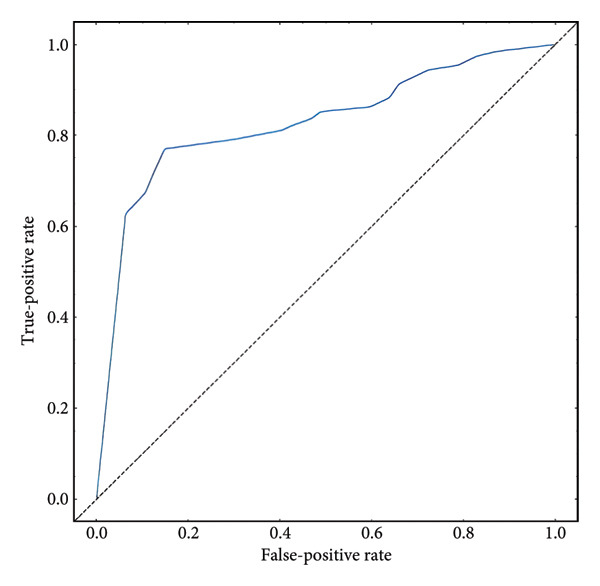
(g)

**Table 5:** Diagnostic performance of the nomogram in the training and validation set.

Group	AUC (95% Cl)	Accuracy	Sensitivity	Specificity
Training set	0.89 (0.82, 0.92)	0.85	0.94	0.87
Validation set	0.85 0.80, 0.90)	0.82	0.92	0.83

Abbreviations: AUC, area under the curve; CI, confidence interval.

## 4. Discussion

Precise characterization of pancreatic lesions is vital for optimizing clinical management, particularly for PDAC. Accounting for approximately 90% of pancreatic malignancies, PDAC is highly aggressive, often metastasizing early and showing limited response to chemotherapy and radiotherapy, leading to a 5‐year survival rate of only about 12% [22, 23]. This grim prognosis places a substantial emotional and psychological burden on patients and their families, making the nurse’s role in symptom management and patient education. For nurse leaders, ensuring rapid and accurate diagnostic pathways is a critical component of quality care management for this vulnerable population.

The pancreas’s specific anatomical relationships present significant diagnostic challenges: The head is adjacent to the duodenum and common bile duct, complicating puncture routes. The body lies deep within the abdomen, surrounded by the stomach and spleen, thereby restricting operative space. The tail lies near the spleen and left kidney, an area rich in blood vessels and lymphatic tissues, increasing the risk of major hemorrhage. These factors complicate biopsy procedures and elevate risks such as bleeding, infection, or tumor seeding [9, 10]. Currently, definitive diagnosis largely relies on endoscopic ultrasound‐guided fine‐needle aspiration. However, this procedure is invasive, prone to complications, and carries a risk of false negatives, with reported accuracy between 81.6% and 91.9% [24].

It is noteworthy that the neoplastic lesions included in this study were exclusively pancreatic epithelial tumors. PNETs, which arise from multipotent stem cells within the pancreatic ductal epithelium, account for less than 5% of all pancreatic tumors [25]. These tumors express neuroendocrine markers and may secrete ectopic hormones [26]. Given their unique clinical presentations, imaging characteristics, and neuroendocrine markers [27], PNETs are relatively easy to differentiate from other pancreatic tumors and were therefore not included in this study.

CT has become the preferred imaging modality for pancreatic tumors due to its high resolution, rapid imaging, widespread availability, and clear depiction of pancreatic anatomy [28, 29]. In comparison, MRI offers superior soft tissue contrast but is limited by longer examination times and higher costs. Positron emission tomography (PET)/CT, although capable of providing metabolic information, is not suitable for large‐scale screening due to its high radiation and expense [30, 31]. Radiomics, a noninvasive diagnostic tool, shows significant promise in improving the diagnosis, staging, and prognosis of pancreatic tumors [32–38]. It notably enhances early detection of small or occult lesions, with studies demonstrating CT‐based radiomic models detecting PDAC with high accuracy, even identifying cases missed by radiologists [32]. Radiomics also aids in staging by assessing tumor resectability and lymph node status [33, 37] and plays a key role in predicting survival and treatment responses, often outperforming traditional methods [34, 35, 38]. Radiomics has also been studied for its ability to differentiate pancreatic lesions. Most previous research has used radiomics to either distinguish pancreatic lesions from normal tissue or to differentiate between two lesion types. Chu *et al.* used CT‐based radiomic features to differentiate PDAC from normal pancreatic tissue with an overall accuracy of 99.2% [39]. Radiomics has demonstrated efficacy in differentiating PDAC from other pancreatic lesions, including inflammatory lesions such as autoimmune pancreatitis and mass‐forming chronic pancreatitis [40, 41], as well as from other tumor types such as PNETs [42]. Building on this, our study pioneers a CT‐based radiomic model for the simultaneous classification of benign, borderline, and malignant pancreatic tumors. This approach overcomes the limitations of conventional imaging methods and establishes a robust framework for the diagnosis, classification, and individualized treatment of pancreatic tumors. Given the recognized variability in optimal lesion contrast, with some lesions appearing best in the arterial phase [43], some showing comparable contrast across different phases [44], we analyzed both arterial and venous phase CT images.

CA19‐9 is the most widely utilized tumor marker for PDAC diagnosis [45]. However, a prospective European study reported that the diagnostic sensitivity of CA19‐9 was only 50%, 29%, and 36% for patients with lag times of ≤ 6, > 6–18, and > 18 months, respectively [45, 46]. While combining CA19‐9 with other methods, such as gene testing, can enhance diagnostic sensitivity [47, 48]. In our study, we observed significantly elevated preoperative CA19‐9 levels in patients with malignant pancreatic tumors compared to those with borderline cases. Based on this, we constructed a diagnostic model integrating radiomics with CA19‐9 for patients with nonbenign pancreatic tumors. This combined model demonstrated superior diagnostic performance, achieving an AUC of 0.85, an accuracy of 0.82, and high sensitivity (0.92) and specificity (0.83). The integration of radiomics and CA19‐9 offers an effective and reliable method for diagnosing pancreatic tumors, capturing not only the spatial heterogeneity and morphological characteristics of the lesions but also their underlying biological properties.

From a nursing perspective, recent studies have highlighted the growing importance of integrating artificial intelligence into clinical workflows to support clinical decision‐making [49]. The early and accurate differentiation of pancreatic lesions can influence nursing priorities, such as preoperative education, psychosocial support, and postoperative monitoring. Incorporating radiomics‐based predictive tools into routine nursing assessment could allow for more personalized nursing interventions. For example, nurse managers could prioritize patients predicted as malignant for expedited diagnostic work‐up and to provide enhanced patient education and psychological support. Conversely, for patients with a low probability of malignancy, the focus would shift to follow‐up scheduling and symptom monitoring. As precision medicine advances, the collaboration between nurses, radiologists, and oncologists will become increasingly essential to ensure that emerging technologies such as radiomics are effectively translated into patient‐centered practice [50].

This study demonstrates significant advantages in the noninvasive diagnosis of pancreatic lesions. First, to the best of our knowledge, this study is among the first to use radiomics‐based ML models for the classification of benign, malignant, and borderline pancreatic tumors, while also exploring the feasibility of combining radiomic features with CA19‐9. Second, this study is based on a large clinical sample (*n* > 500) that comprehensively covers the main pathological types of pancreatic lesions. Third, pretreatment discrimination among benign, malignant, and borderline pancreatic tumors facilitates patient stratification, preventing overtreatment of benign lesions and enabling timely intervention for malignant tumors. Accurate stratification enables nurses to provide more targeted patient education and develop individualized care plans. This model may also serve as a crucial tool within a multidisciplinary team setting, providing radiologists with objective, quantitative data to complement their qualitative reads, and enabling surgeons and oncologists to refine treatment plans.

However, this study has limitations. Its retrospective design may introduce selection bias and restrict generalizability. Besides, radiomics inherently faces challenges, including interobserver variability in segmentation, and the influence of imaging equipment and parameters on model stability [51, 52]. Finally, the absence of an external, multicenter validation cohort also limits the generalizability of our findings. Future external validation with independent patient cohorts is essential to ensure the model’s stability and reliability.

## 5. Conclusions

In this study, we employed CT‐based radiomics analysis to construct ML models that successfully differentiated benign, borderline, and malignant pancreatic tumors, with the RF algorithm exhibiting the best performance. Furthermore, we developed a diagnostic model integrating radiomics and CA19‐9, which exhibited robust and stable diagnostic efficacy as well as clinical utility in identifying PDAC. This model may provide a novel instrument not only for diagnosis and stratification but also for facilitating timely care planning and enhancing the nurse’s role in patient education and advocacy. However, future external validation is needed to ensure the model’s stability and reliability.

**Nomenclature**
ANOVA: Analysis of variance
ANNs: Artificial neural networks
AUC: Area under the curve
CA12‐5: Carbohydrate antigen 12‐5
CA19‐9: Carbohydrate antigen 19‐9
CEA: Carcinoembryonic antigen
CI: Confidence interval
CT: Computed tomography
DCA: Decision curve analysis
GLCM: Gray‐level co‐occurrence matrix
GLDM: Gray‐level dependence matrix
GLRLM: Gray‐level run‐length matrix
GLSZM: Gray‐level size zone matrix
IPMNs: Intraductal papillary mucinous neoplasms
LR: Logistic regression
LASSO: Least Absolute Shrinkage and Selection Operator
MCNs: Mucinous cystic neoplasms
MRI: Magnetic resonance imaging
ML: Machine learning
NGTDM: Neighboring gray tone difference matrix
PDAC: Pancreatic ductal adenocarcinoma
PNET: Pancreatic neuroendocrine tumor
Rad‐Score: Radiomics score
RF: Random forest
ROC: Receiver operating characteristic
ROI: Region of interest
SCNs: Serous cystic neoplasms
SPNs: Solid pseudopapillary neoplasms
SVMs: Support vector machines

## Ethics Statement

This study was approved by the ethics committee of the Affiliated Hospital of Qingdao University (Reference No: QYFY WZLL 28748) and conducted in accordance with the World Medical Association Declaration of Helsinki. Given the retrospective nature of the study, the requirement for informed consent was waived.

## Consent

No individual participant data are reported that would require consent to publish from the participant (or legal parent or guardian for children).

## Conflicts of Interest

The authors declare no conflicts of interest.

## Author Contributions

Xiaoxuan Li designed the study and drafted the manuscript. Jiani Liu contributed to the imaging and clinical data collection. Xinchi Luan performed data analysis. Xiaomin Xing reviewed the literature and performed pathological confirmation. Jinfeng Cui and Shibo Wang revised the manuscript. Jing Guo supervised the data quality control. All authors contributed to the article and approved the submitted version. Xiaoxuan Li and Jiani Liu contributed equally to this work as cofirst authors.

## Funding

This work was supported by the Shandong Provincial Natural Science Foundation (ZR2023QH175).

## Data Availability

The data that support the findings of this study are available from the corresponding author upon reasonable request.

## Appendix 1 The Rad‐Score Formula.

Rad‐Score = diagnostics_Image‐original_Mean∗0.017903665640739173+diagnostics_Image‐original_Minimum∗0.01788276508836534+original_shape_MajorAxisLength∗0.02620353499516506+original_shape_Maximum2DDiameterRow∗‐0.054928112592319174+original_shape_Maximum3DDiameter∗‐0.007444548169657882+original_shape_Sphericity∗0.19963791869320816+original_shape_SurfaceVolumeRatio∗0.1679438962528009+original_firstorder_10Percentile∗0.02073200173097486+original_firstorder_Energy∗0.09433162690817429+original_firstorder_Kurtosis∗‐0.00684654095692284+original_firstorder_Maximum∗0.041875133458725955+original_firstorder_Skewness∗0.0013204649348083001+original_glcm_ClusterShade∗‐0.021916360832497015+original_glcm_Correlation∗0.07100600828585622+original_glcm_Idm∗‐0.18048474814985496+original_glcm_Idmn∗0.09677487864084834+original_glcm_Idn∗0.05796361784898756+original_glcm_Imc1∗0.10837917540938605+original_glcm_InverseVariance∗0.106859878339255+original_glcm_JointEnergy∗‐0.11423320262641021+original_gldm_DependenceEntropy∗‐0.39357391272298103+original_gldm_LargeDependenceLowGrayLevelEmphasis∗0.11042119950662074+original_glrlm_GrayLevelNonUniformityNormalized∗‐0.0530430335960434+original_glrlm_RunEntropy∗0.5321332025807071+original_glrlm_RunVariance‐∗0.0314993622473085+original_glszm_GrayLevelNonUniformity∗‐0.02469923368986767+original_glszm_GrayLevelNonUniformityNormalized∗0.03573376306467116+original_glszm_LargeAreaEmphasis∗0.008456956569385134+original_glszm_LargeAreaLowGrayLevelEmphasis∗‐0.0353877084730159+original_glszm_SizeZoneNonUniformityNormalized∗0.07073802236781089+original_glszm_SmallAreaLowGrayLevelEmphasis∗‐0.027782603699661828+original_glszm_ZoneVariance∗0.006126049147331805+original_ngtdm_Busyness∗0.1268374442068115+original_ngtdm_Coarseness∗0.013006386143299584+original_ngtdm_Complexity∗‐0.003067003358432544+original_ngtdm_Strength∗‐0.06121383717200731.
